# Hypocalcemia in a Saudi intensive care unit

**DOI:** 10.4103/1817-1737.39638

**Published:** 2008

**Authors:** Mobeen Iqbal, Rifat Rehmani, Mohammad Hijazi, Ayman Abdulaziz, Sayed Kashif

**Affiliations:** *Division of Pulmonary and Critical Care Medicine, King Abdulaziz National Guard Hospital, PO Box 2477, Alhasa, 31982, Kingdom of Saudi Arabia*

**Keywords:** Hypocalcemia, intensive care

## Abstract

**OBJECTIVE:**

Hypocalcemia has been a common abnormality in the West, seen in patients admitted to the intensive care unit (ICU). It has also been linked with disease severity. We undertook this study to determine the frequency of hypocalcemia in patients admitted to the intensive care unit.

**MATERIALS AND METHODS:**

In a retrospective chart review from January 2004 till December 2004, patients admitted to our ICU were reviewed. Patients’ age, sex, diagnosis, acute physiology and chronic health score APACHE II and ionized calcium were recorded. Patients were divided into three groups based on disease severity as measured by APACHE II. Hypocalcemia was defined as ionized Ca level less than 1.18 mmol/L. Frequency was determined in each group and correlation of hypocalcemia with disease severity was explored.

**RESULTS:**

Hypocalcemia was seen in 22.2% in group A (APACHE II < 10), 40.4% in group B (APACHE II 10–19) and 53.9% in group C (APACHE II > 19). Hypocalcemia and disease severity (APACHE II scores) were negatively correlated (*P* = 0.02).

Mean ionized Ca levels in groups A, B and C were 1.22 mmol/L (±0.10), 1.19 mmol/L (±0.11) and 1.25 mmol/L (±0.24) respectively.

**CONCLUSIONS:**

Hypocalcemia is a common finding in critically ill patients. It is correlated with worsening disease severity. Mechanisms underlying hypocalcemia and the possible relationship of hypocalcemia with mortality need further consideration.

Hypocalcemia is defined as a reduction in ionized serum calcium (Ca) concentration. In critically ill patients, it is a relatively common laboratory abnormality.[1–2] There are data linking hypocalcemia with increased critical care mortality[1–3] and disease severity,[4–6] though the absolute relationship is not established.[7] Hypocalcemia in critical adult patients has been frequently reported with frequency ranging from 15% to 88%.[4] The cause of hypocalcemia in critically ill patients is multifactorial, including inflammatory cytokines in sepsis syndrome, increased sympathetic activity and impaired calcium homeostasis.[8–9]

The present study was intended to study the frequency of hypocalcemia in patients admitted to a Saudi intensive care unit and whether hypocalcemia has any correlation with disease severity measured by acute physiology and chronic health score APACHE II.

## Materials and Methods

Patients admitted to our ICU over a period of 1 year (Jan 2004–Dec 2004) were screened for inclusion criteria. Intensive care unit at King Abdulaziz National Guard Hospital is 10-bedded and caters to both medical and surgical. Patients admitted from either the emergency department or an operating room having had serum Ca measured within 24 h of admission were eligible for inclusion in the study. The corresponding day arterial blood gas was used for pH and ionized calcium levels. The APACHE II scores were also recorded during the first 24 h of admission. Patients with (1) hospital admission within 2 weeks before presentation (2) supplemental vitamin D therapy at the time of admission (3) known underlying chronic renal insufficiency defined as a creatinine level greater than 2.0 mg/dl (4) preexisting or known parathyroid disease (5) current treatment for malignancy (6) age less than 12 years (7) oral or iv calcium therapy and (8) any blood transfusion before Ca level was drawn were excluded. Three patient groups were defined based on APACHE II scores: group A, patients with APACHE II scores less than 10 (n = 50); group B, patients with APACHE II scores 10–19 (n = 85); and group C, patients with APACHE II scores more than 19 (n = 18). Thus, 153 patients were evaluated.

Patient characteristics include age, sex, admitting diagnosis and preexistent chronic diseases. Parameters for patients included mean arterial pressure, presence of acute renal failure (ARF) and Acute Physiology and Chronic Health Evaluation II (APACHE II) scores at the time of ICU admission. Duration of ICU and hospital stay (days) was recorded as observational data.

Laboratory data included values for total serum calcium (Ca), ionized serum Ca, magnesium (Mg), phosphate, creatinine, albumin and arterial pH. Calcium was analyzed with an ionic-specific electrode (blood gas analyzer) using arterial blood sample. For analysis, pH 7.4 corrected values were used. Hypocalcemia was defined as an ionized Ca level less than 1.18 mmol/L. The normal range for our institution is 1.18–1.33 mmol/L.

To determine frequency of hypocalcemia, patients were considered hypocalcemic if they had ionized Ca level less than 1.18 mmol/L at any time a measurement was available during admission to the ICU for all the groups. The frequency was compared among groups using chi-square test. In addition, absolute Ca levels among groups A, B and C were compared by averaging the multiple Ca levels per patient to arrive at a single value for each patient, namely, the average Ca level.

## Results

Patient characteristics, length of ICU stay, serum magnesium, phosphorus and total calcium in groups A, B and C are shown in Table 1. There was a broad range of admission diagnoses [Table 2] for ICU patients, although the majority of patients in groups A and B were admitted for cardiac problem while sepsis was the commonest admitting diagnosis in group C patients.

**Table 1:** Patient characteristics and laboratory findings

Variables	Group A (n = 50)	Group B (n = 85)	Group C (n = 18)
Age (years)	43.18 ± 15.66	60.6 ± 20.43	68.56 ± 18.87
Sex			
Men	33 (66%)	29 (34.1%)	11 (61.1%)
Women	17 (34%)	56 (65.9%)	07 (38.9%)
Mean length of stay (days)	2.15 ± 1.4	3.27 ± 3.8	4.57 ± 3.5
Serum phosphorus (mmol/L)	1.24 ± 0.32	1.24 ± 0.32	1.24 ± 0.55
Serum magnesium (mmol/L)	0.83 ± 0.16	0.82 ± 0.17	0.85 ± 0.12
Total calcium (mmol/L)	2.06 ± 0.18	2.08 ± 0.19	2.00 ± 0.28

**Table 2:** Distribution of admission diagnosis

Diagnosis	Group A n (%)	Group B n (%)	Group C n (%)
Cardiac	23 (46)	38 (44.7)	2 (11.1)
Pulmonary	6 (12)	15 (17.6)	4 (22.2)
Neurology	4 (8)	7 (8.2)	1 (5.5)
Drug overdose	0	3 (3.5)	0
Gastrointestinal	4 (8)	8 (9.4)	0
Sepsis/septic shock	4 (8)	7 (8.2)	9 (50)
Surgery/trauma/OBGYN	9 (18)	7 (8.2)	2 (11.1)

Frequencies of hypocalcemia, based on ionized calcium, were 22.2% in group A, 40.4% in group B and 53.9% in group C. Frequency of hypocalcemia increased with increasing severity of disease based on APACHE II risk stratification model, which was statistically significant, with *P* = 0.02.

Group A patients had normal mean ionized Ca level of 1.22 mmol/L (±0.10). Among group B patients, the mean ionized Ca level was 1.19 mmol/L (±0.11). Group C patients had mean ionized Ca level of 1.25 mmol/L (±0.24).

There was no correlation found, in our study, of levels of magnesium and phosphate with ionized calcium level.

The mean APACHE II scores for groups A, B and C were 6.8 (±1.62), 13.1 (±2.56) and 23.5 (±3.85) respectively.

We had seven deaths recorded in our cohort, one in group A and 3 each in group B and C. All ICU survivors were alive at the time of discharge or at the end of 30 days in hospital (whichever came first).

## Discussion

Results of our study show that hypocalcemia is very common in critically ill Saudi patients (as high as 54% in our cohort). Progressive decline in calcium levels with increasing disease severity as measured by APACHE II scores is in accordance with the data reported by Zivin *et al.* Due to smaller cohort, correlation of hypocalcemia with mortality could not be concluded. Moreover, magnesium and phosphorus levels did not correlate either with calcium or disease severity in our patients, which makes disease severity a more likely explanation for hypocalcemia.

Several mechanisms have been suggested for hypocalcemia in critically ill patients. These include pro-inflammatory cytokines in septic patients impairing PTH responsiveness,[8–10] catecholamine excess in sick patients, end-organ resistance to PTH, inhibition of PTH secretion and intra- and extracellular redistribution of calcium ion.[11] Hypocalcemia has been reported frequently in ICU setting.[1,2] The question remains open that whether this represents a marker of disease severity or a direct predictor of worse outcome. The frequency of hypocalcemia in our study seems to be within the range reported in literature (15–88%).[4,7]

Correlation of hypocalcemia with mortality is far from proven. In one study, 30-day mortality was not predicted independently by hypocalcemia.[12] It is more likely that hypocalcemia is a marker of disease severity. Moreover, there are no data that treating hypocalcemia can improve mortality in hypocalcemic individuals. On the contrary, calcium replacement may impair myocardial function. In our cohort of patients, there were no deaths reported in ICU survivors at 30 days post discharge from ICU.

This study is the first from the Kingdom to address frequency of hypocalcemia in Saudi intensive care population. Though in our patient population, there is a negative correlation of hypocalcemia with disease severity, this study is underpowered to detect any effect on short-term or long-term mortality. The cause of hypocalcemia in our cohort most likely seems to be due to several mechanisms (e.g. decreased vitamin D3 levels, inflammatory mediators and sepsis). Prospective cohort studies need to be designed to explore the correlation of hypocalcemia with mortality. Moreover, markers of baseline vitamin D deficiency and PTH levels in Saudi patients of variable disease severity need to be studied to explore the possibility of widespread vitamin D deficiency.
